# Research on Robot Grasping Based on Deep Learning for Real-Life Scenarios

**DOI:** 10.3390/mi14071392

**Published:** 2023-07-08

**Authors:** Jie Hu, Qin Li, Qiang Bai

**Affiliations:** 1 College of Big Data Statistics, Guizhou University of Finance and Economics, Guiyang 550025, China; jason.houu@gmail.com; 2 School of Mechanical Engineering, Guiyang University, Guiyang 550002, China; baiqiang@gyu.edu.cn

**Keywords:** hybrid model, robot, grasp, deep learning

## Abstract

The application of robots, especially robotic arms, has been primarily focused on the industrial sector due to their relatively low level of intelligence. However, the rapid development of deep learning has provided a powerful tool for conducting research on highly intelligent robots, thereby offering tremendous potential for the application of robotic arms in daily life scenarios. This paper investigates multi-object grasping in real-life scenarios. We first analyzed and improved the structural advantages and disadvantages of convolutional neural networks and residual networks from a theoretical perspective. We then constructed a hybrid grasping strategy prediction model, combining both networks for predicting multi-object grasping strategies. Finally, we deployed the trained model in the robot control system to validate its performance. The results demonstrate that both the model prediction accuracy and the success rate of robot grasping achieved by this study are leading in terms of performance.

## 1. Introduction

Achieving accurate and efficient grasping is a fundamental requirement for the widespread implementation of robotic systems. Based on human grasping experience, it is evident that a well-designed grasping strategy plays a significant role in achieving a successful grasp, which is essential for the success of a robot’s grasping task. Currently, robot grasping research primarily focuses on two main directions: top grasping with RGB input [1,2,3] and multi pose grasping with RGB-D or point-net as input [4,5,6,7]. Despite the advantage of strong universality, multi-pose grasping relies on 3D point-net, which leads to slower performance and necessitates the continuous observation of the object from various angles to achieve a stable grasping strategy in case of occlusion, thereby limiting the wide application of multi-pose grasp. Conversely, top-grasping based on RGB only needs a top-view angle to perceive the object’s shape and generate appropriate grasping options, which makes it run faster. Furthermore, deep learning-based image processing technology has significantly promoted the advancement of grasping strategy research, thereby expanding the application prospects of top-grasping strategies. One of the mainstream research methods to improve the object detection algorithm is through the generation of rectangle grasping strategies [8,9,10,11,12,13,14,15]. For instance, Joseph Redmon et al. [8] developed an object detection model with a mixture of convolution layer and full connection layer and realized grasp detection and object classification at the same time, with excellent performance in speed and accuracy. Victor Parque et al. [11] proposed an RGB-based grasping attitude prediction model using the GoogLeNet framework. The model can achieve excellent prediction performance under the training of a small number of label datasets. The YOLO series, as a classic algorithm, has made remarkable achievements in the field of object detection. Therefore, researchers have improved the YOLO series to generate grasp strategies: Cosimo Della Santina et al. [12] built an anthropomorphic soft hands grasping model based on YOLOv2 and achieved a grasping success rate of 81.1%; Wafae SEBBATA et al. [15], based on YOLOv3, achieved object recognition and attitude estimation at the same time, achieving a 79.9% success rate of grasping on different objects. With the development of depth cameras and the improvement of hardware level, it has become a research direction to realize 3D point-net reconstruction of the object based on RGB-D and then achieve 6-DOF grasping attitude prediction [16,17,18]. Jens Lundell et al. [16] proposed a generating deep network—DDGC—which realizes the generation of high-precision 6-DOF grasp strategy in complex environments. Korrawa Karunratanakul et al. [17] designed a variable encoding–decoding model based on deep learning for 3D point cloud processing, thereby generating a reasonable grasp pose. Yongxiang Wu et al. [18] proposed an end-to-end pixel level grasp prediction network, which realized the conversion from depth images to point cloud information and achieved 97% and 90% grasp success rates on common and unusual objects, respectively. The traditional object detection algorithm has low accuracy and poor generalization performance. While point cloud-based 6-DOF grasp provides high accuracy, it demands sophisticated hardware and slow model speed. Consequently, research on grasp strategy based on semantic segmentation has emerged as a viable research direction [19,20]. Douglas Morrison et al. [19] proposed semantic segmentation models using two parallel fully connected networks for generating grasping strategies. They achieved success rates of 88% and 83% for common and uncommon objects, respectively. Yong Ho Na et al. [20] used a fully connected network to predict the robot grasping pose based on RGB and achieved success rates of 79% and 74% on the training and test sets, respectively. The aforementioned studies demonstrate that semantic segmentation algorithms based on RGB data can achieve object segmentation at the pixel level without requiring complex point cloud information. Consequently, these algorithms are a promising avenue for research on grasping strategies. However, current research primarily focuses on enhancing object detection algorithms for predicting grasp strategies, which results in poor generalization and low success rates in actual grasp experiments, thereby limiting their practical applicability. To address these issues, this paper proposes a novel hybrid segmentation model that merges a convolutional neural network (CNN) and a residual networks to fully utilize their respective abilities for information extraction and linear transformation. By comparing various semantic segmentation algorithms, the proposed hybrid model makes a valuable contribution to the field. Moreover, to ensure the integrity of the research, the trained model is deployed in a robot operating system for high-precision grasp verification.

Therefore, the novelty of this paper lies in: (1) conducting theoretical analysis on the excellent performance and existing issues of residual networks and designing four improved residual block structures; (2) designing two CNNs and four residual structures based on the actual working conditions of robot grasping and using them as the foundation to construct a hybrid model for robot grasping strategy; and (3) not only completing the training and testing of the hybrid model at the algorithm level but also deploying the model in the robot operate system to achieve the application validation of robot grasping. By combining theoretical research and practical verification, this paper designs a novel grasping strategy prediction model and achieves excellent performance in practical grasping.

This paper is divided into five sections. Section 1 provides a summary and analysis of current mainstream methods, including an evaluation of their advantages and disadvantages. Additionally, this section presents the research direction for this paper. Section 2 introduces the principles of two algorithms and constructs a hybrid model. Section 3 describes the training and testing processes of the model. Section 4 outlines the construction of the robot grasping application platform. The final section provides the conclusions.

## 2. Materials and Methods

This paper aims to construct a model for predicting rectangular grasp strategies by utilizing a combination of CNN and residual networks. By integrating CNN’s semantic information extraction with residual network linear transformation, we aim to achieve pixel-level predictions for rectangular grasp strategies.

### 2.1. Performance Difference of CNN with Different Structures

The emergence of CNN has sparked a global surge in the adoption of deep learning techniques. Central to the architecture of CNNs is the convolutional kernel, which is a digital matrix of a predetermined size. The convolutional kernel’s primary function is to execute a numerical transformation on the input image, enabling the extraction of feature information embedded within the image. The characteristic map value can be determined through the utilization of the following formula:

(1) $$G(m,n)=(f*h)[m,n]=\sum_{i}\sum_{k}h[j,k]f[m-j,n-k]$$

where the input images are marked as *f*, the convolution kernel is marked as *h*, the row and column indexes of the calculation results are marked as *m* and *n*, and *j* and *k* are convolutional kernel sizes.

The convolution operation can be classified into two types: valid convolution and same convolution. In valid convolution, no padding is applied to the input image, resulting in a gradual reduction in the size of the image after each convolution. On the other hand, the same convolution utilizes a padding technique where a specific width of zeros is added around the input image to ensure that the output size is the same as the input size. The padding width is calculated using a specific formula:

(2) $$P=\frac{f-1}{2}$$

where *P* is the filling size and *f* is the convolution kernel size (usually odd).

**Figure 1:**
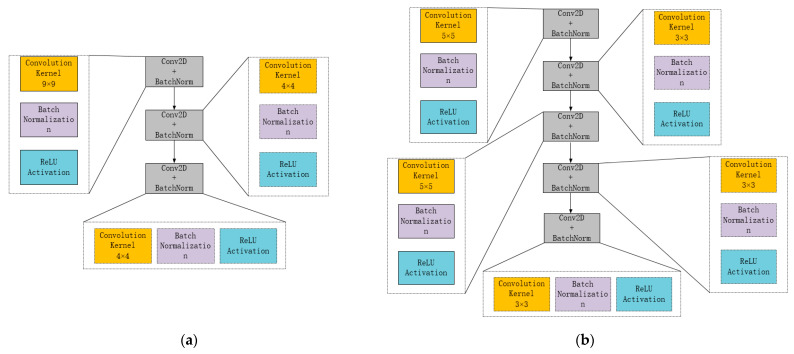
Structure of convolution layer (**a**) shallow large-scale; (**b**) deep small-scale.

To ensure simultaneous acquisition of semantic information and image resolution, it is crucial to choose a reasonable number of convolution layers as more layers will decrease image resolution. The size of the convolution kernel is also an essential parameter of CNN, as it directly affects the efficiency and ability of CNN to gather semantic information. Therefore, this paper will optimize the grasp strategy prediction model by considering the number of convolution layers and the size of convolution kernels while integrating it with the residual networks. After analyzing the model layers and information acquisition ability, two CNN structures were designed (as illustrated in Figure 1).

Figure 1a illustrates that a large convolution kernel provides a broader receptive field, resulting in better global features with more image information. However, this also causes a significant increase in computation, which impedes the depth of the model and reduces its computational efficiency. In contrast, Figure 1b depicts the current prevalent approach of replacing the large convolution kernel with continuous small convolution kernels. This results in more nonlinear activation functions, stronger semantic expression capabilities, fewer parameters, and lower hardware requirements. However, it also introduces new issues like gradient disappearance and over-fitting. To address these concerns, this paper proposes two models with different structures.

### 2.2. Theoretical Analysis of ResNet

**Figure 2:**
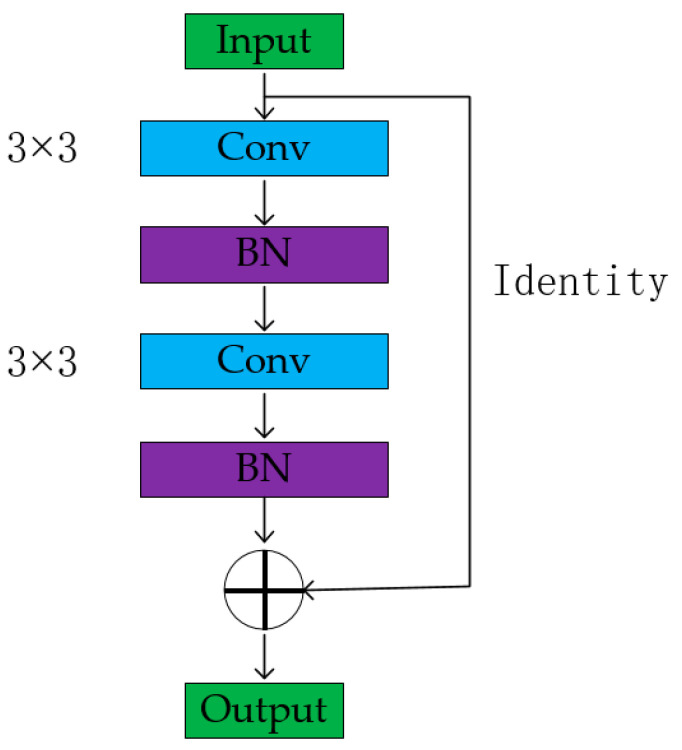
The principle residual block.

From the perspective of the development of deep learning, the depth of the network plays a crucial role in the performance of the model. Increasing the number of network layers allows for the extraction of more complex feature patterns, theoretically leading to better results as the model becomes deeper. However, researchers have found that increasing the depth of the network also results in the following issues: (1) model overfitting, (2) gradient vanishing or exploding, and (3) the significant waste of computational resources. The emergence of ResNet has successfully addressed the long-standing issues of deep learning. ResNet accomplishes this by constructing a standard forward convolution network and incorporating a skip connection that circumvents a specific convolution layer. The combination of the forward convolution network and the skip connection creates a residual module, as illustrated in Figure 2. Deep networks are more vulnerable to performance degradation than shallow networks. When multiple layers within a deep network are transformed into identity maps (*h(x) = x*), the model regresses to a shallow network. However, deep learning is a complex nonlinear mapping network, and it is extremely challenging to directly learn identity mapping. Residual networks introduce a novel approach by structuring the network in the format presented in Figure 2. Additionally, its mathematical explanation is:

(3) H(x)=F(x)+x⇒F(x)=H(x)−x

where *F(x) = 0* is the identity map, and *F(x)* is the residual.

Residual networks provide two solutions to mitigate the issue of declining model performance: identity mapping and residual mapping. Identity mapping corresponds to the straight line depicted in Figure 2, whereas residual mapping relates to the residual part. *F(x)* denotes the network mapping before summation, while *H(x)* represents the network mapping from input to model post-summarization. The integration of the residual module enhances the model’s sensitivity to output fluctuations, augments its ability to adapt to the weight, and culminates in superior results.

From a mathematical point of view, the residual block can be expressed as:

(4) $$x_{l+1}=x_{l}+F(x_{l},W_{l})$$

Through recursion, the expression of *L* characteristics of any deep unit in the network can be obtained:

(5) $$x_{L}=x_{l}+\sum_{i=l}^{L-1}F(x_{i},W_{i})$$

That is, *W* is the weight in convolution calculation, and the characteristics of any deep unit *L* can be expressed as the characteristics *x_l_* of shallow unit *l* plus a residual function in the form of $\sum_{i=1}^{L-1}F$, which indicates that both (*L* and *l*) have residual characteristics.

Similarly, for unit *L* of any depth, its characteristic is:

(6) $$x_{L}=x_{0}+\sum_{i=0}^{L-1}F(x_{i},W_{i})$$

which is the sum of the output of all previous residual functions and is added to *x*_0_ to obtain the final sum.

The loss function *ε* with respect to the gradient of *x_l_* can be expressed using the back-propagation derivative chain propagation rule.

(7) $$\begin{array}{l} \frac{\partial \varepsilon }{\partial x_{l}}=\frac{\partial \varepsilon }{\partial x_{L}}\frac{\partial x_{L}}{\partial x_{l}}=\frac{\partial \varepsilon }{\partial x_{L}}\left(1+\frac{\partial }{\partial x_{l}}\sum_{i=l}^{L-1}F\left(x_{i},W_{i}\right)\right)\\ =\frac{\partial \varepsilon }{\partial x_{L}}+\frac{\partial \varepsilon }{\partial x_{L}}\frac{\partial }{\partial x_{l}}\sum_{i=l}^{L-1}F\left(x_{i},W_{i}\right) \end{array}$$

The above formula reflects two attributes of the residual networks:

(1) In the complete training process of the model, the value of $\frac{\partial }{\partial x_{l}}\sum_{i=l}^{L-1}F\left(x_{i},W_{i}\right)$ will not always be −1, which explains why the gradient in the residual networks does not disappear;
(2) $\frac{\partial \varepsilon }{\partial x_{L}}$ shows that the gradient of layer *L* in the model can be directly transferred to any shallow layer *l* without passing through the weight layer, while $\frac{\partial \varepsilon }{\partial x_{L}}\left(\frac{\partial }{\partial x_{l}}\sum_{i=l}^{L-1}F\left(x_{i},W_{i}\right)\right)$ is transferred through the weight layer.

By examining the forward and backward propagation mechanisms of residual networks, it has been concluded that if a residual block meets the two aforementioned conditions, then information can be efficiently transmitted between the shallow and deep layers, thus making these two conditions sufficient for achieving successful deep model in residual networks.

From the perspective of necessity, it is assumed that:

(8) $$h\left(x_{l}\right)=\lambda_{l}x_{l}$$

where *λ* is the coefficient of identity map in the residual network 

At this time, the residual block can be expressed as:

(9) $$x_{l+1}=\lambda_{l}x_{l}+F(x_{l},W_{l})$$

For deeper *L* layer, the general expression of residual block can be obtained by substituting Formula (8) into Formula (6):

(10) $$x_{L}=\left(\overset{L-1}{\underset{i=l}{\prod }}\lambda_{i}\right)x_{l}+\sum_{i=l}^{L-1}F(x_{i},W_{i})$$

In order to simplify the problem, this study only considers the left half of the Formula (10): $x_{L}=\left(\prod_{i=l}^{L-1}\lambda_{i}\right)x_{l}$, the loss function ε calculates the partial differential of *x*_1_ to obtain:

(11) $$\frac{\partial \varepsilon }{\partial x_{l}}=\frac{\partial \varepsilon }{\partial x_{L}}\left(\left(\prod_{i=l}^{L-1}\lambda_{i}\right)+\frac{\partial }{\partial x_{l}}\overset{\wedge }{F}\left(x_{i},W_{i}\right)\right)$$

The aforementioned formula presents two important attributes. 

Specifically, when λ > 1, the gradient is likely to experience an explosion, while when λ < 1, the gradient is prone to instantaneously change to 0. 

The latter situation can significantly hinder the reverse transfer of data in the residual networks, thus adversely affecting the training process. To mitigate the impact of λ on the model, it is recommended to set its value to 1. 

**Figure 3:**
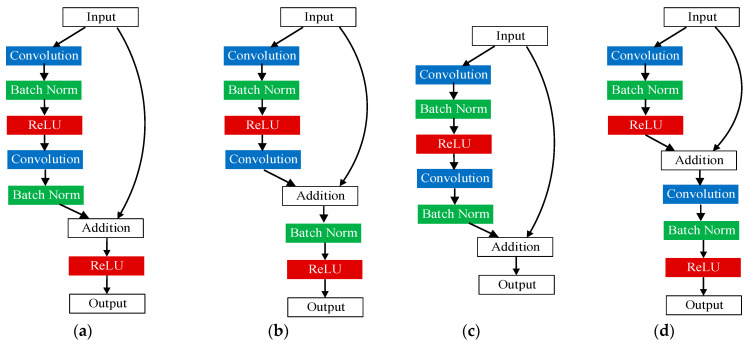
Improved residual block structure (**a**) Long-identity ResNet; (**b**) Short-identity ResNet; (**c**) No-ReLU ResNet; (**d**) Min-identity ResNet.

The fundamental idea underlying the construction of residual networks is to establish the residual between the input and output, which enables the networks to effectively integrate information. The residual block depicted in Figure 3a encompasses several typical convolutions and direct skip connections that link input to output. This strategy of incorporating skip connections into the model constitutes a structure-based normalization technique.

In addition to the representation of the residual block described by Formulas (4) and (9), an alternative expression is also possible:

(12) $$x_{l+1}=\sigma \left(f\left(x_{l},W_{l}\right)+x_{l}\right)$$

In the residual networks, the residual block can be expressed as follows: given an input *x_l_*, *f*(*●*) represents the joint operation of several convolution layers in Figure 3, where *W_l_* represents all the parameters contained in *f*(*●*), and *σ*(*●*)represents a nonlinear operation. The activation function used in residual networks is ReLU, which sets every element less than 0 in the input to 0. Since *σ*(*●*) is a nonlinear function, *f(x_l_*, *W_l_) + x_l_* is not always greater than 0. Therefore, the following inequality can be derived:

(13) $$\sigma \left(f\left(x_{l},W_{l}\right)+x_{l}\right)\neq \sigma \left(f\left(x_{l},W_{l}\right)\right)+\sigma \left(x_{l}\right)$$

The above formula indicates that the nonlinearity introduced by the residual block is responsible for the inadequate learning of the difference between its input *x_l_* and output *x_l_*_+1_ *– x_l_*. Similarly, a residual network composed of multiple residual blocks may encounter difficulties in effectively learning the difference between the input *x*_0_ and the output *x_L_* of the entire network.

This study investigates the impact of the nonlinear transformation in residual networks on the model’s performance, with a focus on normalization (BN) and activation function. To address this issue, the paper proposes residual blocks with different structures based on the blocks presented in this work and evaluates their performance. Four residual network structures, shown in Figure 3, are utilized, and experiments are conducted to verify their performance differences. Specifically, Figure 3b places batch normalization after the addition operation, which speeds up model training and reduces the risk of gradient vanishing but may cause data distortion and negatively affect model accuracy. Figure 3c eliminates the ReLU layer behind the conventional residual block to avoid altering the identity transformation of the data. Finally, Figure 3d incorporates ReLU into the skip connection to mitigate the semantic information loss caused by the nonlinear transformation of the convolution layer to the image, thus enhancing model accuracy.

### 2.3. Overall Structure of Hybrid Model

**Figure 4:**
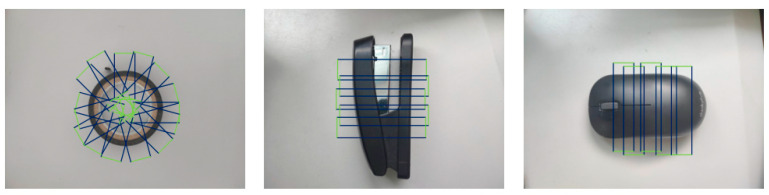
Dataset visualization. Green-Dexterous hand;Blue-The itinerary of a dexterous hand.

The Cornell Grasp Dataset is used both as the training and testing set in this paper. The dataset contains labels representing valid grasping positions in the form of grasp rectangles, as shown in Figure 4. This dataset is a crucial resource for implementing deep learning-based autonomous robot grasping and contributes significantly to the development of this field. Many advanced vision-based robot grasping models have been trained and tested using this dataset. To increase the number of grasping samples, data augmentation techniques are utilized, resulting in 1035 images and 240 objects. The number of grasping samples is increased to 51,000 via random cropping, scaling, and rotation. Data augmentation plays a vital role in enhancing the prediction performance and generalization ability of the model. The paper sets the training-to-testing ratio to 8:2 to ensure adequate training.

Currently, a limited number of researchers utilize residual networks for grasp strategy prediction [21,22,23,24,25,26,27,28], but their research differs from the content of this paper. Table 1 presents recent papers on robot grasping research based on ResNet and reveals that they are all based on existing models and do not involve improving the core structure of ResNet. This paper analyzes the impact of different sizes and numbers of convolutional kernels on CNN performance and constructs two different CNN structures. It also analyzes the principle of residual blocks, which is the skip connection, and designs four different architectures of residual blocks. Finally, it constructs a mixed model of multiple CNN and ResNet structures to conduct research on robot grasping strategies.

**Figure 5:**
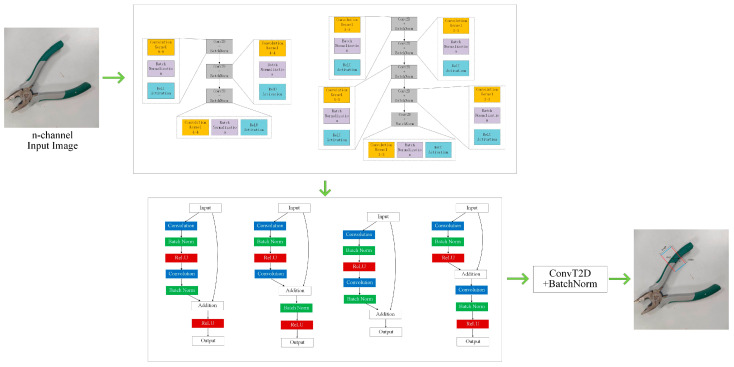
Overall architecture of the model.

This paper presents a deep learning model that integrates convolution structure and residual block to generate a rectangle grasp strategy. The model consists of general convolution, residual, and inverse convolution layers, as shown in Figure 5. These layers extract semantic and policy information and perform up-sampling. Previous performance analysis has shown that different CNN and ResNet structures produce varying results. Therefore, this study combines these structures to filter and achieve an accurate grasp strategy.

## 3. Results

The convolution and residual modules constitute the central framework of the network, and the efficacy of their structures significantly affects the model’s performance. This section aims to optimize the model based on the architecture proposed in Section 2. Table 2 outlines the precise structure of the model proposed in this study, and the performance evaluation is conducted by incorporating the enhanced CNN and ResNet into diverse model configurations. Due to the imprudent nonlinear transformation in the residual structure depicted in Figure 3b, the accuracy of the model is prone to decrease. As a result, the B and F structures in Table 2 are disregarded.

### 3.1. Experimental Environment

The training environment is the 64 bit of Ubuntu 18.04, which adopts the pytorch deep learning framework. The hardware configuration is as follows: core i9-9900x, RAM 128 GB, NVIDIA GeForce RTX2080Ti*2.

### 3.2. Combination of Large Convolution Kernel and Residual Networks

**Figure 6:**
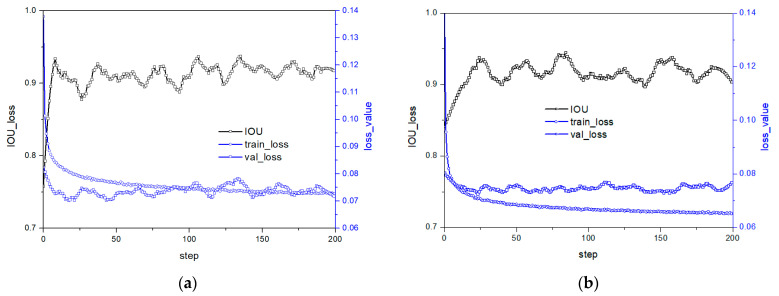
Model performance under structure A in Table 2; (**a**) no-dropout [23] (**b**) dropout 0.5.

A in Table 2 presents the model framework proposed by Sulabh Kumra et al. [9]. The model inputs an N-dimensional image and outputs three pixel-level grasp options, namely grasp quality, grasp angle, and grasp width. The model achieves this output through a process that involves passing the image through three convolution layers and five residual layers, followed by a convolution transposition layer that generates four images: grasp quality score, cos and sin values of the grasp angle, and grasp width. The grasp angle range is subsequently converged to [−π/2, +π/2] by fusing the cos and sin values. The model achieves an accuracy of 97.7% and 96.6% for image and object, respectively. However, the model’s performance in IOU training and prediction fluctuates significantly, indicating that its stability is poor. As IOU is crucial to the rationality of the grasp strategy, the model’s weak generalization performance is mostly due to over-fitting, as revealed by the loss and predicted value of the model on the test set that fluctuate greatly, while the loss curve on the test set rebounds slightly with increasing number of iterations. Figure 6a illustrates the training and prediction results of the model. This figure is the previous work of the authors of this paper [23], and this method only combines the large convolution kernel and classical residual. In this section the comprehensive performance of CNN and residual networks with different structures and the deployment of the optimal model on robot for grasping experiments will be presented. 

To alleviate over-fitting, this paper proposes using dropouts to enhance the model’s generalization performance. The analysis of Figure 6b suggests that while dropout has some effect on stability and accuracy, its impact is limited. This implies that the model’s structure itself may be flawed and cannot be improved solely through the implementation of dropout.

**Figure 7:**
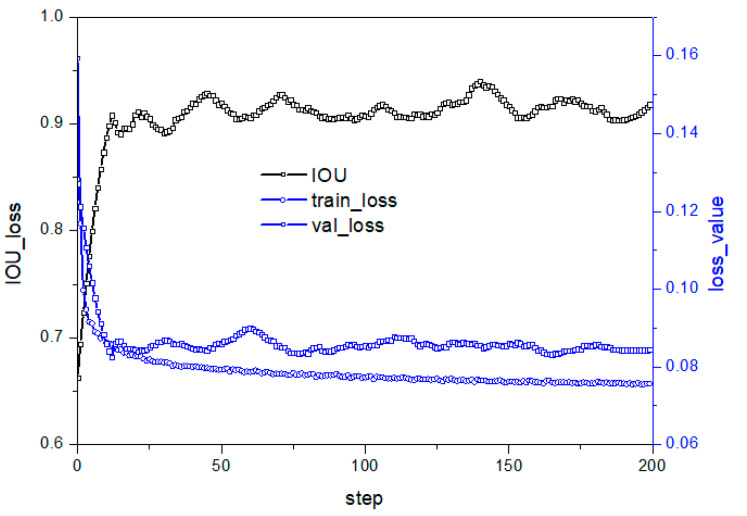
Performance under C structure in Table 2.

The activation function is crucial in deep learning to stimulate hidden nodes and introduce nonlinear features. ReLU is a popular activation function, but its problem of a negative value of 0 reduces model performance. This study aims to improve the activation layer to enhance performance. Figure 7 illustrates the loss and prediction curve under the model structure in C in Table 2. Removing the ReLU layer leads to a well-suppressed loss curve fluctuation on the test set, indicating better convergence towards the optimal value. Reducing nonlinear transformation at a specific position in the residual network improves model stability. However, the IOU prediction curve shows that the test set model stability still slightly improves but fluctuates significantly, with a slightly lower accuracy rate than Figure 6b, due to the reduced semantic information extraction ability after reducing the nonlinear change structure.

**Figure 8:**
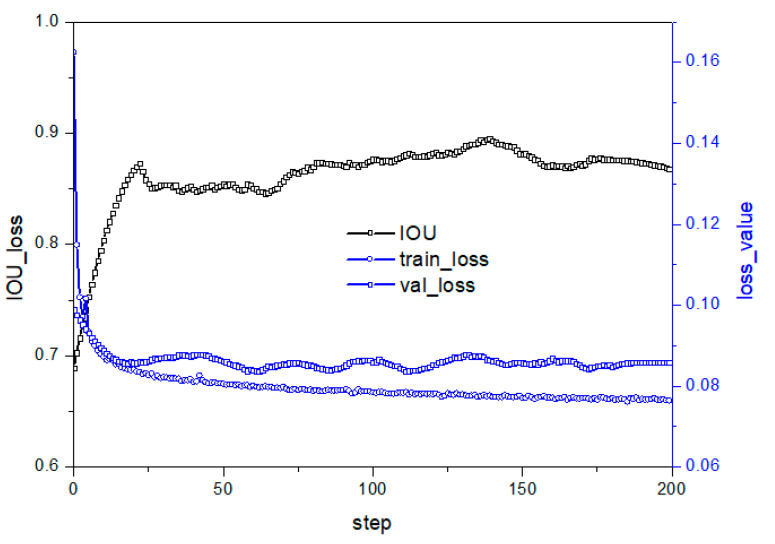
Performance under D structure in Table 2.

The skip connection is the central aspect of residual networks, effectively solving the problem of information loss due to the convolution layer’s nonlinear transformation of the image. To further enhance residual networks, this study proposes advancing the skip connection’s position to the ReLU layer, as shown in Figure 3d. This modification helps the neural network preserve more image information and reduces the risk of overfitting. Figure 8 illustrates the improved model’s loss and performance curve, demonstrating reduced model volatility after adjusting the skip connection to the ReLU layer. However, the model’s performance on the test set remains poor due to the nonlinear transformation position’s significant impact on semantic segmentation accuracy and stability. Therefore, further improvements are necessary to achieve both objectives.

Our research shows that the improved residual module significantly reduces the model’s performance fluctuation on the test set. However, beyond a certain number of iterations, the model’s loss value and accuracy no longer improve due to the model’s insufficient semantic information extraction ability. To address this issue, we propose improving the CNN connected to the residual networks to enhance the model’s performance. Figure 5 illustrates the convolution structure divided into 9 × 9–4 × 4–4 × 4 and 5 × 5–3 × 3–3 × 3–3 × 3–3 × 3. While the 9 × 9–4 × 4–4 × 4 convolution structure used previously increased the receptive field and extracted more information to obtain better features, it also resulted in higher computational requirements and was not conducive to deeper model structures. Previous studies have shown that using several smaller size convolution kernels achieves better performance [29,30], such as the VGG model. Therefore, this paper proposes changing the model’s convolution kernel from a three-layer 9 × 9–4 × 4–4 × 4 to a five-layer 5 × 5–3 × 3–3 × 3–3 × 3–3 × 3–3 × 3 structure to improve the model’s semantic information extraction ability while alleviating the residual networks’ shortcomings. To ensure the study’s integrity and systematicness, this paper proposes three improved model structures of convolution layers (EGH in Table 2).

### 3.3. Combination of Small Convolution Kernel and Residual Networks

**Figure 9:**
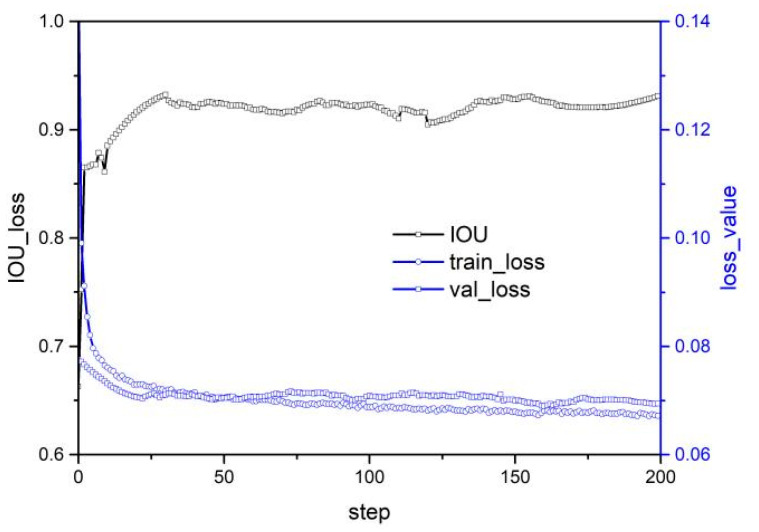
Performance under E structure in Table 2.

**Figure 10:**
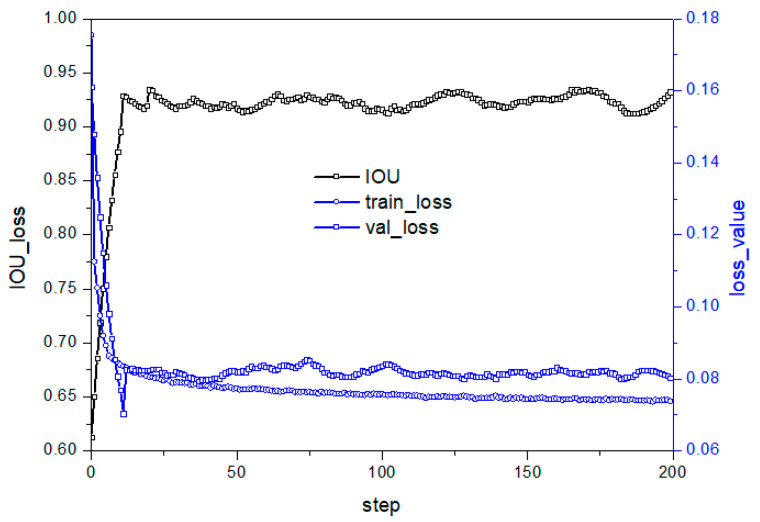
Performance under G structure in Table 2.

Figure 9 illustrates the training and test curves of E in Table 2. Reducing the convolution kernel to 5 × 5–3 × 3–3 × 3–3 × 3–3 × 3 from 9 × 9–4 × 4–4 × 4 improves the stability of both loss and predicted values. Increasing the convolution layer allows the model to obtain more semantic information, thereby enhancing feature fitting. Figure 10 shows the training and test curves of G in Table 2, which exhibits stable but poor accuracy due to the reduced nonlinear transformation. A five-layer CNN before the residual layer alleviates accuracy problems caused by insufficient information extraction. Compared with Figure 8, the model in G in Table 2 has lower loss values, indicating enhanced fitting ability. Although the fitting ability of the G structure is slightly lower than that of E, the generalization ability has improved, leading to good performance in the test set. 

**Figure 11:**
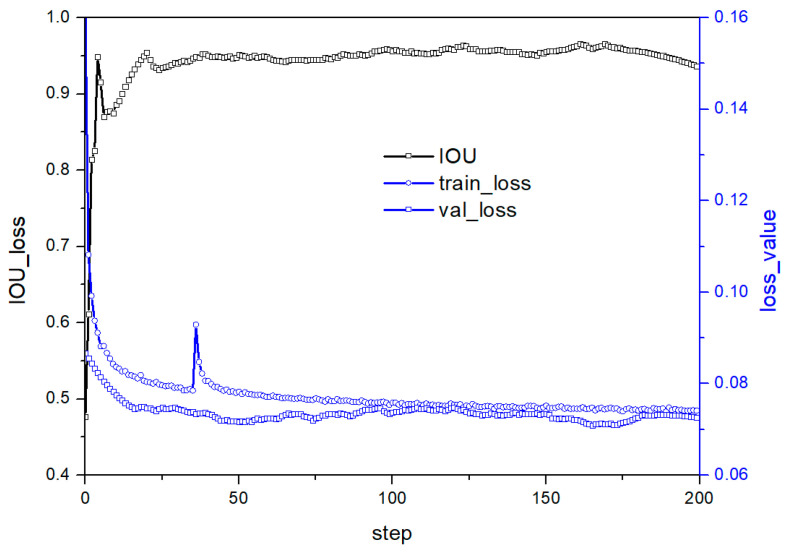
Performance under H structure in Table 2.

To obtain the H structure in Table 2, we combine the convolution module of 5 × 5–3 × 3–3 × 3–3 × 3–3 × 3 with the structure in Figure 3d to form a new residual network. This network advances the skip connection to the end of the ReLU, integrating more information before the convolution layer and completing the prediction of the grasp strategy after further information extraction through the convolution layer. Figure 11 illustrates the loss and prediction curve of the H structure. The model’s accuracy continues to improve steadily with the increase of iterations after advancing the ReLU layer. The loss value steadily decreases, indicating significantly improved fitting ability. The prediction curve shows that the model has excellent stable and strong generalization performance with gradually improving accuracy as the number of iterations increases without major fluctuations.

### 3.4. Further Optimization of Parameters

The integration of CNN and residual networks has significantly improved stability and accuracy through the improvement of the model structure. In this study, we optimize the model hyperparameters based on the optimal architecture (H in Table 2). Hyperparameters can be manually adjusted before or during training and are divided into three types: network parameters, optimization parameters, and normalization parameters. Since the network parameters have been optimized, we focus on optimizing the optimization and normalization parameters. Increasing the batch size can reduce training time and improve stability, but it can also reduce the model’s generalization ability if it exceeds the critical value. If the batch size is too small, it can result in lengthy and inefficient training. Assuming a batch size of 1, each training iteration involves a single datapoint. When dealing with a large dataset (e.g.: 100,000 pieces of data), the model needs to be fed with data 100,000 times. Completing a full pass through the data would take a significant amount of time, resulting in low training efficiency. Additionally, a small batch size can make it difficult for the model to converge, leading to underfitting. In the case of batch size 1, training with individual data introduces significant parameter variations due to differences between individual instances or the influence of outliers. This high randomness in gradients at each layer requires a considerable amount of time and makes it challenging for the model to converge. Therefore, increasing the batch size moderately greatly benefits model training, but it also imposes higher hardware requirements. A larger batch size reduces training time while requiring more memory capacity. With the same number of epochs, a larger batch size reduces the number of batches needed, resulting in faster processing speed and shorter training time. However, if the batch size is too large, for instance, batch size = 100,000, attempting to feed all 100,000 data into the model at once may cause memory overflow. Moreover, while a larger batch size enhances stability, it may also lead to a decline in the model’s generalization ability. Within a certain range, increasing the batch size promotes convergence stability, but as the batch size continues to increase, the model’s generalization performance tends to deteriorate. If the batch size is set to the maximum value (equal to the total number of samples), each parameter update would be based on the same set of samples, resulting in a nearly deterministic descent direction. This scenario would adversely impact the model’s generalization performance.

**Figure 12:**
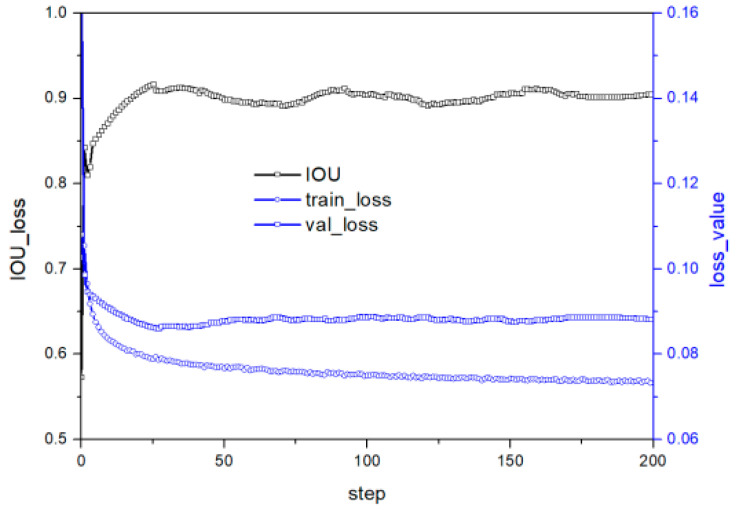
The loss and performance curve after adjusting the batch size from 32 to 64.

Therefore, we increase the batch size from 32 to 64 after referring to relevant papers [31,32]. Figure 12 shows the loss and performance prediction curve of the model after adjusting the parameters. The model’s stability does not change significantly after the batch size increases. However, the loss curve on the test set increases instead of decreasing with the increase of iterations, and the prediction performance slightly decreases. This is because increasing the batch size alone increases the risk of over-fitting and reduces the generalization of the model, which is not conducive to improving model performance.

Based on previous research and relevant papers [33,34], this study modifies the optimization algorithm from SGD (stochastic gradient descent—SGD is a gradient-based optimization algorithm used to update the parameters of deep neural networks) to Adam (Adam uses momentum and adaptive learning rate to accelerate the convergence speed) while maintaining the same batch size. The major drawback of SGD is its slow convergence and susceptibility to local optima, which can significantly affect model performance. To address these issues, Adam, an integrated optimization algorithm, is proposed. First-order momentum is added to SGD to obtain SGD-M, while AdaGrad and AdaDelta add second-order momentum to SGD. Adam is developed by combining both first and second-order momentum.

The first-order momentum of SGD is

(14) $$m_{t}=\beta_{1}\times m_{t-1}+(1-\beta_{1})\times g_{t}$$

and the second-order momentum of AdaDelta is

(15) $$V_{t}=\beta_{2}\times V_{t-1}+\left(1-\beta_{2}\right)\times g_{t}^{2}$$

The two most common hyperparameters ($\beta_{1}$ and $\beta_{2}$) in the above formula, and the former controls the first-order momentum and the latter controls the second-order momentum.

**Figure 13:**
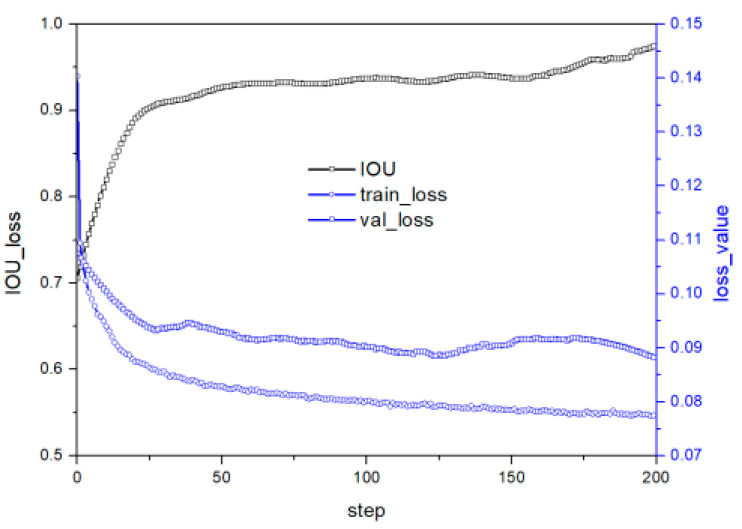
Change the optimization algorithm from SGD to Adam based on Figure 12.

Figure 13 illustrates the loss and performance curve of the model following the optimizer modification. Despite a slight rebound in the loss value, the model demonstrated outstanding prediction performance on the test set. These findings indicate that Adam exhibits favorable optimization performance, attributable to its ability to merge the benefits of Adarad and RMSprop when dealing with sparse gradients and non-stationary objects. Furthermore, Adam computes distinct adaptive learning rates for each parameter, facilitating faster iterations at the initial training stages, followed by a gradual reduction in the learning rate as the optimal value is approached, thereby enhancing the model’s stability.

## 4. Discussion

### 4.1. Model Prediction Results

The previous sections discussed various improvements and optimizations that have significantly enhanced the stability and accuracy of the model. This section aims to provide an objective and intuitive evaluation of the model’s performance, including stability and visualization. Table 3 presents a detailed overview of the performance of hybrid networks with different structures, indicating that the model’s overall performance steadily improves with each epoch. An epoch refers to each complete cycle of forward and back propagation of the model for all data during the training process. The model’s fitting ability to the training set gradually improves with an increase in epoch, indicating continuous performance improvement. However, exceeding a certain epoch value can lead to over-fitting, causing a decline in generalization performance. The specific epoch value needs to be determined based on comprehensive consideration of the model’s complexity and the dataset size. In this paper, an epoch value of 200 was selected after conducting several experiments.

To ensure a systematic and comprehensive study, this paper further enhances the model’s performance by optimizing hyperparameters and conducting depth optimization regarding batch size and optimizer. The analysis of the data presented in Table 3 indicates a significant improvement in the model’s prediction performance following parameter optimization.

Table 3 demonstrates an improvement in the model’s performance through the optimization and adjustment of the number and size of convolution kernels and the location of nonlinear transformations. Initially, the model’s convolution kernel was adjusted from a 9 × 9–4 × 4–4 × 4 to a 5 × 5–3 × 3–3 × 3–3 × 3–3 × 3 structure. The smaller convolution kernels increase the number of layers of the model, which improves the nonlinear expression ability. Additionally, small convolution kernels have more convolution times, which means stronger feature extraction capabilities. Moving the skip connection forward allows the model to comprehensively utilize shallow layer and low complexity features, resulting in a smooth decision function with better generalization performance. Therefore, the H structure in Table 2 has optimal performance.

**Figure 14:**
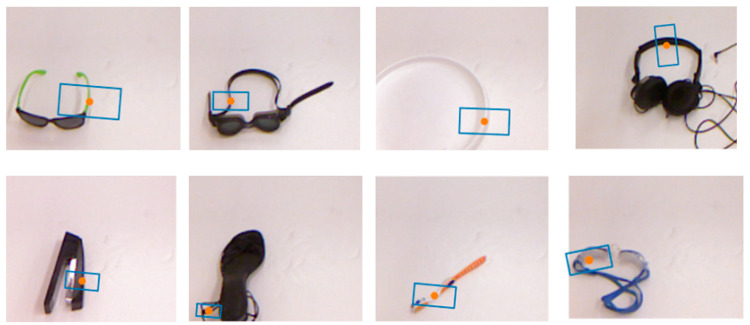
Data visualization results: the first row is the success of prediction, and the second row is the failure of prediction.

Figure 14 presents the results of the predictions, displaying successful and failed grasp strategies. The model successfully predicts appropriate grasp strategies for irregular and circular objects. The center point represents the central coordinate of the grasp strategy, the short side refers to the gripper side, the long side indicates the gripper’s travel distance, and the rectangle’s angle represents the gripper’s grasp angle. The results reveal that a reasonable grasp angle has a significant impact on the success rate, and failure strategies are primarily due to the following reasons: First, the unreasonable position of the grasp is one of the important reasons for the grasp’s failure (such as stapler and high-heeled shoes). Secondly, inappropriate grasping angles can also lead to grasping failure (such as toothbrushes and goggles).

Table 4 provides a detailed breakdown of the prediction performance of various models, facilitating comparison between the proposed model and previous research. The results reveal that some existing research achieves high algorithmic accuracy but lacks application verification or does not translate well to actual grasp accuracy. For instance, the full convolution network [35] of Elias De Coninck et al. achieves impressive theoretical prediction accuracy but experiences a significant drop in success rate during actual object grasping due to the study’s use of synchronous motion between the camera and manipulator, which increases the difficulty of image processing. On the other hand, the emergence of semantic segmentation algorithms has enabled researchers to integrate them into grasp strategy generation. In 2021, Mingshuai Dong et al. [22] propose a two-step grasp strategy prediction model that utilizes a residual networks as the backbone. This model first segments the object to be grasped from the image and subsequently predicts the grasp strategy accurately based on the segmentation results. Although this approach is generalizable, it is yet to be verified through real-world applications. In the field of machine vision, object detection and semantic segmentation are the two primary research areas. However, object detection relies on fitting processes based on label data and cannot learn contour features, which significantly impacts grasp success. This provides an explanation for the observed gap between model prediction accuracy and actual grasp accuracy.

### 4.2. Application Validation

The aim of the present study is to deploy the grasping strategy prediction model to the robot control system to achieve the object grasping task. This deployment involves communication between the computer and the robot, and various issues such as coordinate and pixel point conversion. To provide empirical evidence for the theoretical results of this paper and promote the development of the robot grasping field, this section examines multi-object grasping using the open source robot operate system (ROS) for robot control. For this purpose, the Baxter robot produced by Rethink Robotics is employed as the verification platform. The Baxter robot’s joints integrate elastic brakes and have springs between the motor/transmission device and the actuator. This configuration enables real-time monitoring of external forces and enables it to work alongside humans in a practical environment. Table 5 presents the specific parameters of the Baxter robot used in this study.

**Figure 15:**
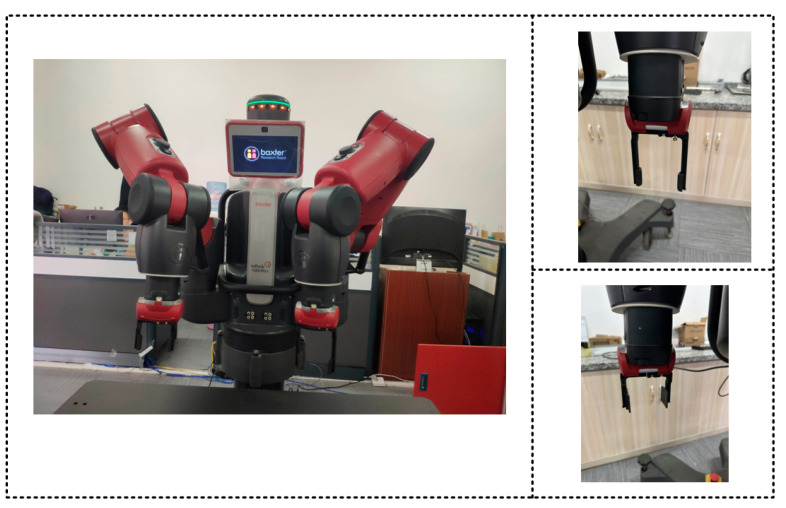
Picture of a Baxter robot.

Robots are extensively used in industrial settings, but there are few instances of collaborative work between humans and robots. This is because mainstream manipulators lack cooperative capabilities, and human–machine collaborations pose a potential danger to human safety. Figure 15 illustrates the overall structure of the Baxter robot, which features two 7-DOF mechanical arms on the left and right. The Baxter robot facilitates the deployment of models and robot motion control in this study by supporting the open-source SDK based on Linux. The robot also comes equipped with two two-finger grippers with varying widths, which greatly aid in object grasping.

**Figure 16:**
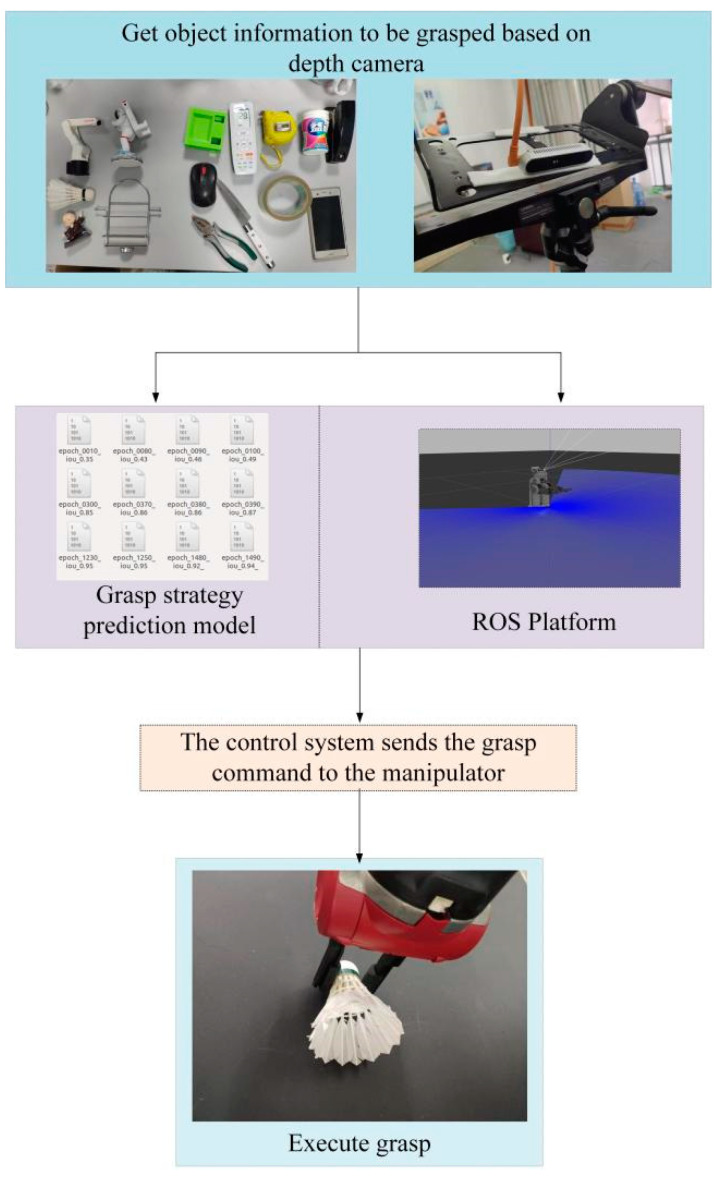
Object grasp process of two-finger dexterous hand.

Figure 16 illustrates the detailed process of the actual grasping procedure. Figure 16 illustrates the detailed process of the actual grasping procedure. The main phases are divided into the following parts. (1) Deployment of the trained grasp strategy model to the ROS platform. Firstly, a workspace is created and compiled, followed by the installation of the camera driver. Next, the model is placed in a specified path, and finally, the deployment is completed by configuring the model runtime environment and adding execution permissions. (2) The camera obtains images of the object for grasping. The images are stored in a designated directory, and the model reads these images got by the camera, performing processing and prediction on them. (3) The model predicts the optimal grasp strategy and executes it. Based on the images acquired by the camera, the model generates multiple grasp strategies and ranks them according to a scoring system, ultimately selecting the best strategy as the final grasp strategy. (4) The localization of the object is achieved through ROS. Firstly, the positions of the camera, object, and robotic arm are determined using hand–eye calibration and camera calibration, establishing the correspondence between 2D and 3D coordinates. Then, utilizing the OpenCV module within ROS, the coordinates of the central pixel point are determined. (5) The robotic arm performs the grasping action. This paper utilizes the Baxter robot, which is supported by a Linux-based open-source control system. Hence, the motion control of the robotic arm is implemented using the MoveIt module within ROS. Finally, the robot receives the grasp command, enabling the robot arm to approach the object and complete the grasping operation.

### 4.3. Result Analysis

**Figure 17:**
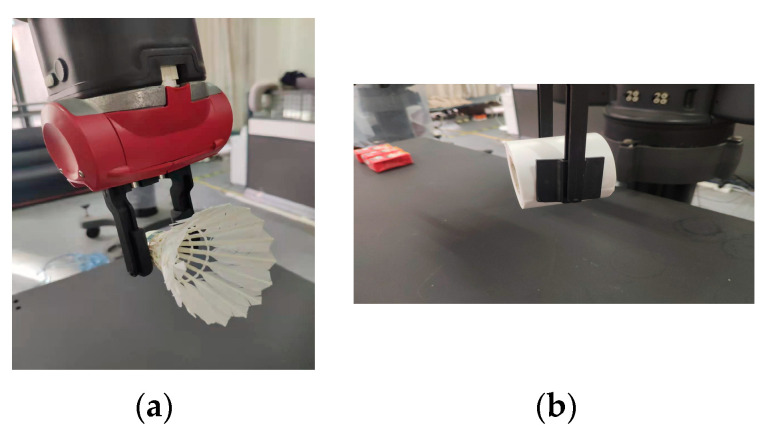
Schematic image of successful grasping: the upper row shows the narrow gripper; the lower row shows the wide gripper. (**a**) Example of successful grasp of narrow griper; (**b**) Example of successful grasp of wide gripper.

In this investigation, 15 distinct objects were chosen for the purpose of conducting grasp experiments. These objects include both regular and irregular items that are commonly encountered in daily life. To verify the generalization ability of the model, grasp experiments were conducted on various types of objects. Figure 17 illustrates instances of successful grasping, where the upper row presents the narrow edge gripper, and the lower row represents the wide edge gripper. It should be noted that the two-finger gripper has unique characteristics, such that blindly increasing the contact area is not a viable strategy for improving the success rate when the object shape is irregular. This is because it would not only fail to increase the success rate, but it would also compromise the stability of grasping. Therefore, when the object shape is complex, this study employs the narrow edge gripper to perform accurate grasping, which Improves the success rate. Each object was grasped 10 times, resulting in an overall success rate of 90%. The success rate for common objects was 93%, whereas for uncommon objects, it was 84%. On the other hand, this paper analyzes the success rates of the dexterous hands in grasping objects of different softness, hardness, and sizes. Firstly, due to the significant deformations observed in soft objects compared to almost negligible deformations in hard objects, the grasping success rate for hard objects (95%) is noticeably higher than that for soft objects (87%). Drawing from human grasping experiences, it is widely known that smaller objects are generally more challenging to grasp than larger ones. This observation aligns with the results obtained in this study, where the grasping success rate for large objects was 94%, while for small objects, it was 85%.

Table 6 presents a detailed and objective comparison of the performance of different algorithms in actual grasping. The results indicate a significant difference in performance among the algorithms, primarily attributable to the hardware capabilities of the manipulator. For instance, the four-finger gripper used by Zhao Zengzhi et al. [41] shows a markedly higher success rate compared to other types of grippers. This is due to its multi-joint design, which offers excellent encapsulation but slower grasping speed. Regarding overall accuracy, the proposed model’s performance is not optimal. This is because other models generally predict and verify the grasping of common objects with established shape rules. However, the proposed model is tested not only on typical life objects but also on irregularly shaped objects, leading to a reduced grasp success rate. Observing the model’s success rate separately on common objects shows that it achieves a leading success rate. However, on uncommon objects, the success rate is significantly lower due to the difficulty of grasping such objects.

## 5. Conclusions

Accurately grasping objects is a crucial precondition for the wide-scale deployment of robots, and therefore represents a prominent research direction for scientists around the world. This paper proposes a semantic segmentation model that leverages CNNs and residual networks to investigate the fundamental problem of object grasping, namely, grasp strategy. The theoretical analysis conducted in this study provides a foundation for enhancing CNN and residual networks, constructing mixed models with different structures, and evaluating their performance.

The experimental results indicate that the size and number of convolution kernels, as well as the number and connection position of nonlinear transformations in the residual networks, significantly affect the model’s performance. Specifically, small convolution kernels, deep networks, and skip connections integrated into the activation function, are conducive to improving the model’s stability. Moreover, changing the optimization function from SGD to Adam led to a significant improvement in model performance.

The proposed model achieved 98.5% prediction accuracy in the test set and a 90% grasp success rate on the robot. These results demonstrate the effectiveness of the proposed approach in addressing the problem of object grasping. The optimal architecture achieved excellent performance in both the training and testing sets, indicating its potential for practical application in real-world scenarios.

This research expands the field of robot grasping and broadens its application by providing a novel approach for addressing the problem of grasp strategy. The proposed model leverages the strengths of both CNNs and residual networks, which are widely used in computer vision and deep learning applications. The model’s accuracy and stability have been enhanced through theoretical analysis and practical experimentation, demonstrating its potential for widespread application.

In conclusion, this paper proposes a semantic segmentation model that leverages CNNs and residual networks for object grasping strategy prediction. The model has been evaluated on a real robot platform, demonstrating excellent performance in both accuracy and stability. This research provides a valuable contribution to the field of robot grasping and promotes the development of collaborative work between humans and robots. In future work, this model could be further enhanced and applied in real-world scenarios to address the challenges of object grasping.

## Figures and Tables

**Table 1:** Object grasp papers.

Year	Author	Model	Model Accuracy (%)	Experimental Accuracy (%)
2017	Ludovic Trottier [21]	C-ResNet	89.15	-
2021	Mingshuai Dong [22]	MASK-GD	96.8	-
2020	Sulabh Kumra [25]	GRCNN	95.4	93
2022	Maomao Shan [26]	CSP-ResNet	96.8	95.6
2022	Juntong Yun [24]	ResNet	90.67	87.33
2021	Zheyuan Hu [27]	TC-ResNet	96.5	-
2023	Sheng Yu [28]	CGNet	92.9	91.7
2022	Li Shaobo [23]	C-ResNet	94.5	-

**Table 2:** The structure of model.

A	Conv 9 × 9	Conv 4 × 4	Conv 4 × 4			
	BatchNorm	BatchNorm	BatchNorm	ResNet (a)		
	ReLU	ReLU	ReLU			
B	Conv 9 × 9	Conv 4 × 4	Conv 4 × 4			
	BatchNorm	BatchNorm	BatchNorm	ResNet (b)		
	ReLU	ReLU	ReLU			
C	Conv 9 × 9	Conv 4 × 4	Conv 4 × 4			
	BatchNorm	BatchNorm	BatchNorm	ResNet (c)		
	ReLU	ReLU	ReLU			
D	Conv 9 × 9	Conv 4 × 4	Conv 4 × 4			
	BatchNorm	BatchNorm	BatchNorm	ResNet (d)		
	ReLU	ReLU	ReLU			
E	Conv 5 × 5	Conv 3 × 3	Conv 5 × 5	Conv 3 × 3	Conv 3 × 3	
	BatchNorm	BatchNorm	BatchNorm	BatchNorm	BatchNorm	ResNet (a)
	ReLU	ReLU	ReLU	ReLU	ReLU	
F	Conv 5 × 5	Conv 3 × 3	Conv 5 × 5	Conv 3 × 3	Conv 3 × 3	
	BatchNorm	BatchNorm	BatchNorm	BatchNorm	BatchNorm	ResNet (b)
	ReLU	ReLU	ReLU	ReLU	ReLU	
G	Conv 5 × 5	Conv 3 × 3	Conv 5 × 5	Conv 3 × 3	Conv 3 × 3	
	BatchNorm	BatchNorm	BatchNorm	BatchNorm	BatchNorm	ResNet (c)
	ReLU	ReLU	ReLU	ReLU	ReLU	
H	Conv 5 × 5	Conv 3 × 3	Conv 5 × 5	Conv 3 × 3	Conv 3 × 3	
	BatchNorm	BatchNorm	BatchNorm	BatchNorm	BatchNorm	ResNet (d)
	ReLU	ReLU	ReLU	ReLU	ReLU	

**Table 3:** Performance comparison under different structures.

	Method (%)	944 + ResNet (a)	944 + ResNet (c)	944 + ResNet (d)	53,333 + ResNet (a)	53,333 + ResNet (c)	53,333 + ResNet (d)	Adam—SGD, Epoch: 32–64
Epoch								
10		89.89	88.70	80.41	88.54	89.59	88.64	79.78
20		89.89	90.87	86.67	91.60	93.45	95.42	83.15
30		91.01	89.11	85.34	93.24	91.96	94.06	86.64
40		86.52	91.92	84.97	92.05	91.95	95.11	88.76
50		94.38	92.00	85.41	92.37	91.72	95.15	91.01
60		83.15	90.60	85.09	91.84	92.39	94.67	92.13
70		93.26	92.80	85.73	91.52	92.69	94.46	94.36
80		94.38	91.50	87.02	92.35	92.28	94.61	95.03
90		95.51	90.52	87.09	92.37	92.26	95.18	95.45
100		88.76	90.76	87.63	92.27	91.63	95.67	95.68
110		92.13	91.08	87.95	91.00	92.06	95.32	95.47
120		92.13	90.69	88.20	90.48	93.02	95.81	95.5
130		93.26	91.80	88.64	91.37	92.99	95.64	95.5
140		89.89	93.97	89.30	92.61	91.92	95.51	96.52
150		88.76	91.67	88.12	92.81	92.34	95.55	96.63
160		92.13	91.57	87.14	92.59	92.55	96.28	97.43
170		88.76	92.29	87.12	92.07	93.36	96.44	97.75
180		95.51	91.22	87.63	92.08	92.20	95.65	98
190		94.38	90.39	87.36	92.45	91.41	94.82	98.32
200		89.89	91.82	86.81	93.09	93.25	93.67	98.5

**Table 4:** Performance comparison between different models.

Author	Year	Representation	Algorithm	Accuracy (%)		Speed (ms)
				Model	Experiment	
Elias De Coninck [35]	2020	rectangle	—	99	90	
Mingshuai Dong [22]	2021	rectangle	MASK-GD	96.5	—	9.43
Eduardo Godinho Ribeiro [36]	2021	rectangle	single-stage regression	90.9	85.7	8.51
François Lévesque [37]	2018	rectangle	—	—	84	—
Weiwei Shang [38]	2020	rectangle	CNN + MDN	—	95	—
David Liu [39]	2023	rectangle	Digital Twin (DT)-CycleGAN	90	85	—
Hamidreza Kasaei [40]	2023	rectangle	DL	92.6	90	—
Ours	2023	rectangle	C-ResNet	98.5	—	8.78

**Table 5:** Baxter robot parameters.

Joint	Maximum Speed (Radian/s)	Bending Stiffness	Peak Moment
S0	2.0	843 Nm/rad	50 Nm
S1	2.0	843 Nm/rad	50 Nm
E0	2.0	843 Nm/rad	50 Nm
E1	2.0	843 Nm/rad	50 Nm
W0	4.0	250 Nm/rad	15 Nm
W1	4.0	250 Nm/rad	15 Nm
W2	4.0	250 Nm/rad	15 Nm
Total weight (kg)	135.2	Maximum power (W)	720
Maximum grasp force (N)	35	Maximum load (including gripper) (kg)	2.2
Position accuracy (mm)	±5 mm	DOF	7 × 2
Operate system	ROS		

**Table 6:** Comparison of grasp experiment results.

Method	Common Object Accuracy (%)	Uncommon Object Accuracy (%)	Overall Accuracy (%)	Number of Fingers
Model-based object grasp [37]	-	-	84.1 (673/800)	Rectangle (Two)
Mixed density grasp prediction network [41]	-	-	95 (57/60)	Rectangle (Four)
Demo-based object grasp [42]	-	-	91.34	Rectangle (Two)
Autonomous grasp [36]	-	-	85.7	Rectangle (Two)
David Liu [39]	-	-	85	Rectangle (Two)
Hamidreza Kasaei [40]	-	-	90	Rectangle (Two)
Ours_rectangle_no_sponge	89 (89/100)	78 (39/50)	85.3	Rectangle (Two)
Ours_rectangle_with_sponge	93 (93/100)	84 (42/50)	90	Rectangle (Two)

## Data Availability

The data presented in this study are available on request from the first author.
